# Progress of Medical Science in Germany

**Published:** 1875-03-01

**Authors:** Edmund J. Doering


### PROGRESS OF MEDICAL SCIENCE IN GERMANY.

By Edmund J. Doering, M.D.

### RELAPSING FEVER.

DR. LAPTSCHINSKI, who has made a series of microscopical examinations of the blood of patients suffering with relapsing fever, reports among others, the following interesting case, embodying the very important conclusions the author has arrived at, as the result of his investigations.

The patient, a robust, well-developed young man, twenty-one years of age, was admitted into the hospital on the fifth day of the disease, with a temperature of 105.5° in the evening. On the following day, with a temperature of 105°, some blood was drawn by the author, mainly with the hope of finding the confervæ discovered by Obermeier in the blood of patients with relapsing fever, for which the author had searched in vain in all the cases examined by him thus far. But again he was disappointed; for neither on this day, nor on the next, nor at any time during the second paroxysm, could he find any confervæ.

At the very first examination, the author was astonished in finding not only an enormous increase of the white blood-corpuscles, but also a great accumulation of colorless cells, which could easily be distinguished from the former by their great size and irregular appearance. Some of these cells contained also fatty matter. On the addition of acetic acid, one or several nuclei, surrounded by granules, could be seen in their interior. These were evidently granular cells from the spleen, which have been discovered previously by Prof. Ponfick in the blood of relapsing fever patients, and have been described by him.

Although the relative proportion of the white to the red blood-corpuscles, previous to the present illness, was not known to the author, still he sees no reason to suspect any abnormal relation to have existed, the patient being a stout, hearty man, never having suffered from any serious illness.

On the eighth day of the disease, the blood-corpuscles were counted, the paroxysm of fever having abruptly terminated the night previous by a copious perspiration, with a fall of temperature from 105.5° to 96°. The proportion was 1 white to 64.3 red corpuscles. According to all appearances, the number of white corpuscles was even greater on the two previous days.

During the apyrectic interval, the number of white corpuscles gradually diminished, as was verified by repeated counting.

Thus, on the eleventh day of the disease, the proportion of white to red corpuscles was 1:98; on the thirteenth day, 1:119.5; on the morning of the fourteenth day (the relapse commencing in the evening), 1:138.

On the following day, the proportion rose at once to 1:41.2, and remained about stationary (1:50.8 and 1:37.5); falling again with the termination of the paroxysm.

The following table explains itself:

| Days of Disease. | Blood-Corpuscles. Red. | | White. | | Proportion. | Temperature. A.M. | P.M. |
|---|---|---|---|---|---|---|---|
| 8 | 3219 | : | 50 | = | 64.3 : 1 | 96° | 96° |
| 11 | 3528 | : | 36 | = | 98.0 : 1 | 98.5 | 98.6 |
| 13 | 3705 | : | 31 | = | 119.5 : 1 | 98.3 | 98.4 |
| 14 | 4972 | : | 36 | = | 138.1 : 1 | 98.1 | 103.6 |
| 15 | 5069 | : | 123 | = | 41.2 : 1 | 103. | 105. |
| 17 | 3862 | : | 76 | = | 50.8 : 1 | 104. | 104. |
| 18 | 4430 | : | 118 | = | 37.5 : 1 | 104. | 96. |
| 19 | 4966 | : | 80 | = | 62.0 : 1 | 96.8 | 98.2 |
| 21 | 4759 | : | 54 | = | 88.1 : 1 | 98.2 | 98.3 |
| 22 | 5012 | : | 37 | = | 135.5 : 1 | 98.3 | 98. |
| 24 | 5006 | : | 25 | = | 200.2 . 1 | 98.2 | 98.3 |
| 26 | 5195 | : | 29 | = | 179.1 . 1 | 98.2 | 98.6 |
| 28 | 6613 | : | 38 | = | 174.0 : 1 | 96.8 | 98.3 |

The direct microscopic examination, and the repeated counting of the blood-corpuscles consequently proved that a sudden increase of the white corpuscles occurred during the paroxysm of fever, with a corresponding, but more gradual decrease during the apyrectic interval.

The author could easily demonstrate by the daily examination of the blood, that the diminution of the white corpuscles during the remission, corresponded with a decrease of the number of granular cells, but without a corresponding increase of the detritus found in the blood in very small quantities. As transitions from one form into another could also not be detected, the author is at present unable to explain how these granular cells disappear.

During the second paroxysm, these cells re-appeared in the blood in large numbers, gradually disappearing again after the decline of the fever.

At every paroxysm, the spleen was found to be greatly increased in size.

On the basis of these examinations, the author has arrived at the conclusion, that during the beginning of every paroxysm in relapsing fever, the contents of the spleen are discharged into the blood. If at the same time pathological products enter the blood, constituting perhaps the direct cause of the fever, is a question requiring further investigation.

In conclusion, the author states, that so much is certain, that the confervæ are not constantly to be found in the blood, and thus cannot hold any relation to the pathogenesis of relapsing fever. — *Centralblatt f. d. Med. Wissensch.*, No. 3, 1875.

---

Modern Medical Treatment.— An editorial article in a British cotemporary closes with the following thoughtful words: Our treatment has assumed a character too decidedly stimulant, and not quite sufficiently nutritive. Stimulants ought to be regarded as auxiliaries to nutrition more than they are at present. Nutritive material, as milk, meat-juice, eggs, and various forms of starch, ought to form a greater matter in the dietary of the sick than stimulants, whether nitrogenized or alcoholic; such materials, when assimilated, give supplies of force. Stimulants may assist in their assimilation, and do so; but in themselves, stimulants only furnish limited supplies of force-bearing material. They are, however, a means by which the system may reach some of its physiological reserve fund. Such use may be advantageous or pernicious, according to circumstances; and an ill-regulated or excessive process of stimulation may give results as disastrous, as a wise and intelligent resort to stimulants may be beneficial in its consequences. —*Phil. Med. and Surg. Reporter.*

